# Quantitative Study of Liver Magnetic Resonance Spectroscopy Quality at 3T Using Body and Phased Array Coils with Physical Analysis and Clinical Evaluation

**DOI:** 10.1371/journal.pone.0122999

**Published:** 2015-04-16

**Authors:** Li Xu, Shiyong Gu, Qianjin Feng, Changhong Liang, Sherman Xuegang Xin

**Affiliations:** 1 Department of Radiology, Guangdong Provincial Traditional Chinese Medicine Hospital & postdoctoral mobile research station of Guangzhou University of Traditional Chinese Medicine, Guangzhou, Guangdong Province, People’s Republic of China; 2 Biomedical Engineering School of the Southern Medical University, Guangzhou, Guangdong Province, People’s Republic of China; 3 Department of Radiology, Guangdong General Hospital, Guangzhou, Guangdong Province, People’s Republic of China; 4 Bernard and Irene Schwartz Center for Biomedical Imaging, New York University School of Medicine, New York, New York, United States of America; Shenzhen institutes of advanced technology, CHINA

## Abstract

This study aims to investigate the quality difference of short echo time (TE) breathhold 1H magnetic resonance spectroscopy (MRS) of the liver at 3.0T using the body and phased array coils, respectively. In total, 20 pairs of single-voxel proton spectra of the liver were acquired at 3.0T using the phased array and body coils as receivers. Consecutive stacks of breathhold spectra were acquired using the point resolved spectroscopy (PRESS) technique at a short TE of 30 ms and a repetition time (TR) of 1500 ms. The first spectroscopy sequence was “copied” for the second acquisition to ensure identical voxel positioning. The MRS prescan adjustments of shimming and water suppression, signal-to noise ratio (SNR), and major liver quantitative information were compared between paired spectra. Theoretical calculation of the SNR and homogeneity of the region of interest (ROI, 2 cm×2 cm×2 cm) using different coils loaded with 3D liver electromagnetic model of real human body was implemented in the theoretical analysis. The theoretical analysis showed that, inside the ROI, the SNR of the phase array coil was 2.8387 times larger than that of body coil and the homogeneity of the phase array coil and body coil was 80.10% and 93.86%, respectively. The experimental results showed excellent correlations between the paired data (all *r* > 0.86). Compared with the body coil group, the phased array group had slightly worse shimming effect and better SNR (all *P* values < .01). The discrepancy of the line width because of the different coils was approximately 0.8 Hz (0.00625 ppm). No significant differences of the major liver quantitative information of Cho/Lip2 height, Cho/Lip2 area, and lipid content were observed (all *P* values >0.05). The theoretical analysis and clinical experiment showed that the phased array coil was superior to the body coil with respect to 3.0T breathhold hepatic proton MRS.

## Introduction

Hepatic MRS is an evolving technique with potential capability for improving the diagnostic accuracy of tissue characterization. *In vivo* proton MRS has been applied to several areas of clinical liver research, including investigations on cirrhosis and hepatitis, diagnosis of malignancies, and treatment monitoring. The acquisition of high-quality hepatic proton spectra is technically demanding. In spite of the facts that few investigators have assessed the contribution of both TR and magnetic field strength ^1^H-MRS for hepatic fat quantification [1, 2], hepatic proton MRS technique is still in the early stages of development. The issues of spectral quality and quality assessment are neglected in the literature of hepatic proton MRS. No consensus on the concepts or detailed criteria of quality assessment for MR spectra has been reached among experts [3–6]. The diagnostic value is directly related to the quality of abdominal MRS, which relies on adequate technical factors, such as prescan adjustments of shimming and effective water suppression [3,5]. Linewidth is important for model fitting; bad resolution easily leads to meaningless results in short TE spectra. Strong resonance signals in prescan from the hydrogens in water molecules may interfere with the signals from the lower concentration compounds of interest. The water signal may be suppressed to better discern the resonance signals of the compounds of interest [7–9].

These aforementioned quality perspectives of hepatic MRS have inherent relationship with radio frequency (RF) coil, an essential hardware component in MRS system, accounting for the transmission of RF signal pulses to excite the tissues being interrogated and/or receive the relaxed RF signals from the body tissues. RF coil serves as the immediate interface between the complex chain of MRS hardware and the patient; hence, its performance characteristics are crucial in determination of image quality as measured by the SNR related to the receiver coil and the radio frequency field homogeneity related to the transition coil. The body coil and phased array coil are two kinds of widely-used coils for clinical applications in MRS. The body coil, which is usually integrated inside the gantry of the MRI system, is a kind of volume coil that can be used as a transition coil or receiver coil. The phased array coil is a kind of surface coil array that is usually used only as a receiver coil. For hepatic MRS in clinics, some studies have employed the body coil, whereas others have used the phased array coil. Although differences on the spectral quality in using these two different coils have been noticed, no careful study has been implemented to quantitatively explore such differences [10–12].

The phase array coil is a specially designed coil for receiving RF signals of MRI on the surface closer to the target organ under assessment. The SNR of a surface coil is better than that of a body coil. However, in this study, we try to quantitatively evaluate the quality of breathhold MRS, exploring a compromise between the SNR and spatial resolution of the two commonly used coils in clinics. This study aims to answer the following questions: 1) Are there any significant differences among shimming, water suppression, and SNR using different RF coils?; 2) Are there any significant differences on the major liver metabolite concentrations of Cho/Lip2 height, Cho/Lip2 area, and lipid content using different RF coils?; and 3) Which kind of coil is more suitable for 3.0T hepatic proton MRS?

This study includes two parts, namely, computational electromagnetic simulation and clinical study. The computational electromagnetic simulation is implemented to theoretically obtain the differences on the homogeneity and SNR of the ROI in the 3D liver electromagnetic model. The clinical study includes comparison of the hepatic MRS prescan adjustments of shimming and water suppression, SNR, and hepatic major metabolites content. The result of the theoretical calculation is consistent with that of the clinical study. Various aspects regarding to the quality assessment and quantitative information of MRS with respect to different coils are also analyzed.

## Materials and Methods

### Part 1 *In vivo* comparison of hepatic MRS using body and phased array coils

#### Subjects

The study was approved by the Ethics Review Board of Nanfang Hospital. Written informed consent was obtained from all patients. Twenty patients (11 men, 9 women; age range, 21–65 years; median age, 33 years) with no history of liver disease and normal liver function test results on the evaluation of nonhepatic disease or fatty liver were included in this study.

#### MRS protocol

The clinical experiments were performed using a 3.0T scanner (GE Signa Excite HD; GE Medical Systems, Milwaukee, WI, USA) equipped with a body coil for RF transmission/receiving and a torso phased array coil for receiving. The first spectroscopy sequence (torso phased array coil was used as receiver) was “copied” for the second acquisition (body coil was used as receiver) to produce identical voxel positioning.

**Fig 1:**
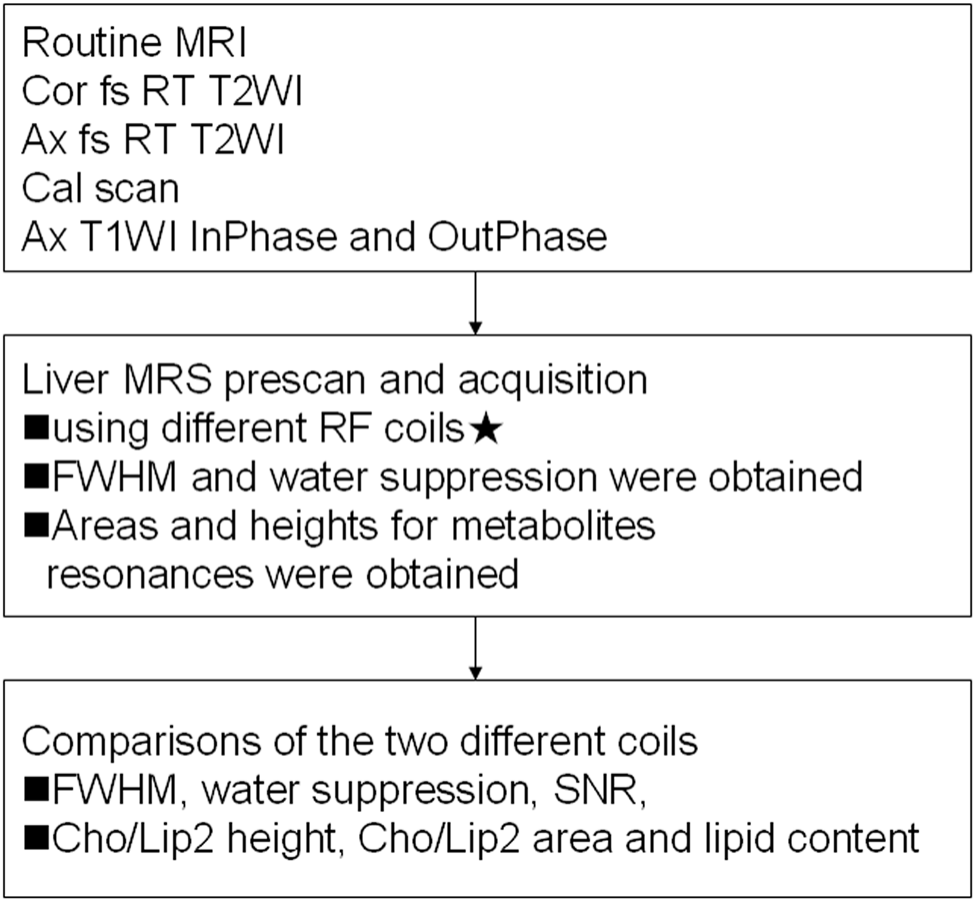
Detailed scanning protocols of hepatic MRS. RT = respiratory triggering; ★ the first spectroscopy sequence (phased array coil was used as the receiver) was “copied” for the second acquisition (body coil was used as the receiver) to produce identical voxel positioning.

The ROI of 20mm×20mm×20mm was positioned in the right hepatic lobe, avoiding the inclusion of the diaphragm and edges of the liver, as well as the vascular and biliary structures. Single-volume spin-echo PRESS was used with parameters of 1500/30/64 (TR/TE/excitations) in all patients. We observed signs of motion artifact on the imaging acquisitions and performed image subtractions of the last imaging sequence at the end of the study from the initial imaging sequence (obtained just before spectroscopic measurements) to discern whether the patient may have shifted in position during the examination. Patients who exhibited motion were excluded; 20 patients were included for the analysis. Detailed scanning protocols are shown in Fig 1. For all data acquisition, water suppression was performed using a series of three chemical-shift-selective pulses with predefined flip angles to produce a significant amount of residual water in the spectrum; high-order shim followed by automatic local shim adjustment were also used. Line widths [full-width half-maximum (FWHM)] and water suppression were obtained.

After acquisition, data were processed using the MR spectroscopic analysis package provided by the MR system manufacturer (SAGE 7.1; GE Medical Systems). The raw data were zero-filled once, apodized with a 5 Hz Gaussian filter, Fourier transformed, and then phase and baseline corrected. Marquardt curve fitting was performed using a Gaussian line shape to calculate the area under the peak. MR spectroscopic data were analyzed by a single medical physicist (L.X.) with more than 7 years of experience in MR spectroscopic analysis. For each MRS measurement of unsuppressed water, we normalized the amplitude of the lipid signal to the sum of the lipid plus water signals to obtain the lipid percentage within the liver.

**Fig 2:**
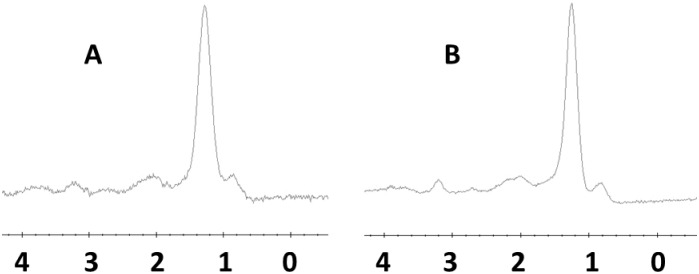
PRESS-localized single voxel 1H MR spectrum originating from liver parenchyma of the same ROI using body coil (a) and phased array coil (b); SNR: (b) was better than (a), so the score was marked 1.

The paired spectra obtained by two coils from the same ROI were compared (assuming A is the spectrum resulting from the phased array coil and B from the body coil). Blind evaluation of the paired spectra was conducted by two radiologists, and the SNR was recorded. When A was similar to, better than, or worse than B, the score was marked 0, 1, or −1, respectively (Fig 2).

#### Statistical analysis

For all tests, a *P* value of less than 0.05 was considered to indicate a statistically significant difference. Statistical analyses were performed with SPSS software (version 10.0.1; SPSS, Chicago, IL, USA). *Mann-Whitney U tests or paired t-test* were used for comparisons of FWHM, water suppression, SNR, Cho/Lip2 height, Cho/Lip2 area, and lipid content between the group using body coil and the group using a torso phased array coil.

### Part 2 Calculation of homogeneity and SNR of the body and phased array coils for theoretical analysis

**Fig 3:**
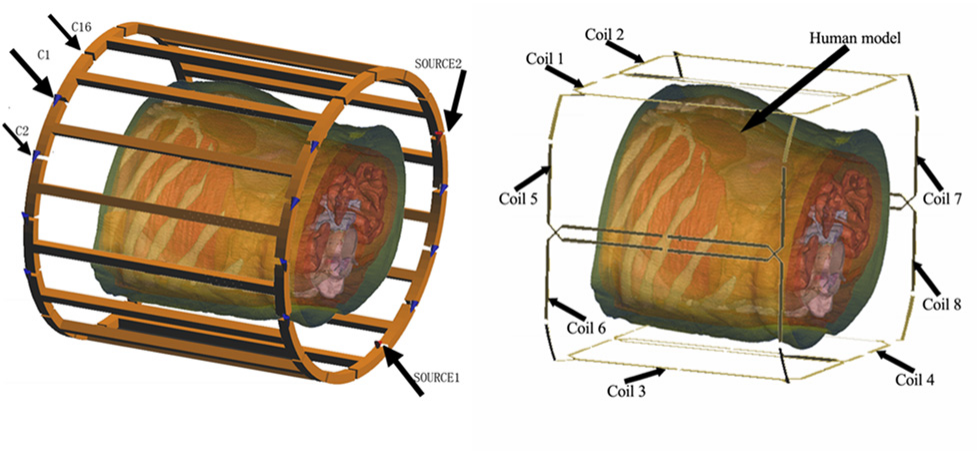
The 16-leg high pass body coil and the eight-element receive phased array coil loaded with human 3D liver electromagnetic model including fourteen different tissues.

First, a 3D computational model was established by manual segmentation of different tissues in all the obtained images of a real human body. After assignment of the dielectric properties of the different tissues (http://niremf.ifac.cnr.it/tissprop/) to the reconstructed 3D model, the final 3D electromagnetic liver model was established. The phased array with eight elements and the body coil with 16 legs, loaded with electromagnetic liver model, are shown in Fig 3. The B_1_ field of the RF coil loaded with the electromagnetic liver model was calculated using the finite difference time domain solver [13, 14]. In this study, the ROI was set as 2cm×2cm×2cm positioned in the right hepatic lobe. The reciprocity theorem was used to analyze the homogeneity and SNR inside the ROI of both body coil and phase array coil [15, 16].

The *SNR* can be calculated as

$$SNR\propto \sin\left(\gamma V\left|B_{1}^{+}\right|\tau \right)\sqrt{B_{1}^{-*}R^{-1}B_{1}^{-}}$$ (1)

where *γ* is the gyromagnetic ratio, *V* is the excitation voltage, *τ* is the duration of the excitation pulse, $B_{1}^{-}$ is the reception field, ***R*** is the n×n noise resistance matrix. The less the *RSD* means the more homogeneous B_1_ field of the transmission field. The homogeneity of $B_{1}^{+}$ field inside the ROI can be represented by the relative standard deviation (*RSD*, *homogeneity* = 1-*RSD*) [16], and calculated as follows:

$$RSD\;=\;\frac{\kappa }{\mu }\times 100\%$$ (2)

where *κ* is the standard deviation of ${|B}_{1}^{+}|$ in the ROI, and *μ* is the mean value of ${|B}_{1}^{+}|$ in the ROI.

## Results

### Part 1 *In vivo* comparison of hepatic MRS using body and phased array coils

A spectrum of normal hepatic parenchyma is displayed as an example in Fig 2.

Significant high positive correlations were observed between the paired data (all *r* >0.86) (Table 1).

**Table 1:** Comparison of hepatic MRS prescan and metabolites content using phased array coil and body Coil (*n* = 20).

	r	P	phased array coil (min-max, median)	body coil (min-max, median)	Z of t value	P
FWHM	0.863	<0.001	17.972.1Hz	17.17z.2Hz	2.223	0.039
Water suppression	0.942	<0.001	92.42..0%	91.91..4%	1.339	0.197
Cho/Lip2 height	0.934	<0.001	0.00–0.50, 0.13	0.00–0.04, 0.07	0.920	0.357
Cho/Lip2 area	0.891	<0.001	0.00–0.42, 0.01	0.00–0.30, 0.01	0.296	0.767
Lipid content	0.971	<0.001	0.00–0.50, 0.02	0.00–0.49, 0.02	0.992	0.321

**Fig 4:**
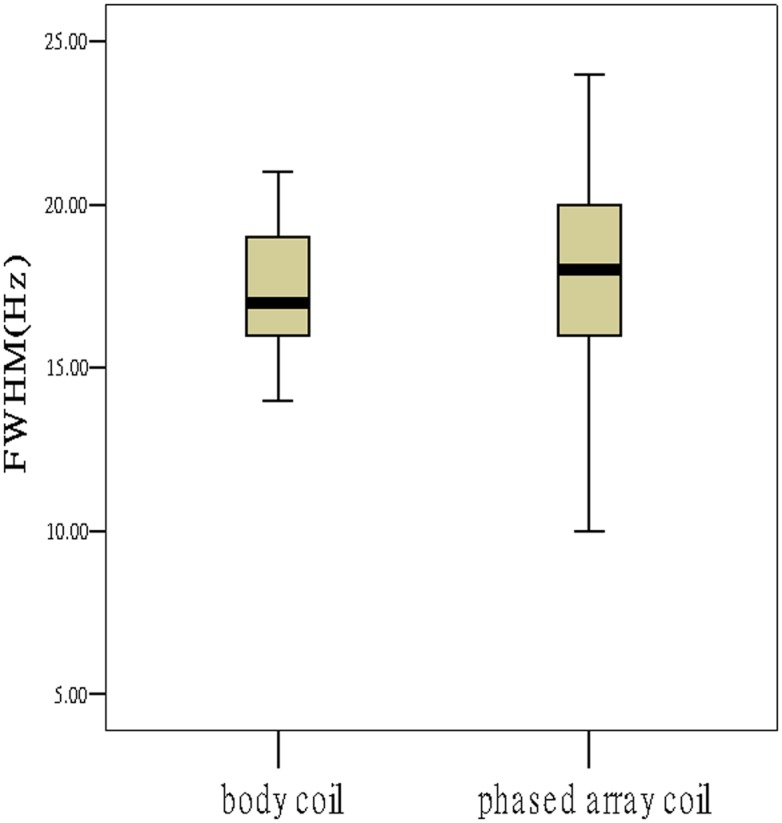
The phased array group had slightly worse shimming effect.

Comparing the paired spectra from the two coils, the group using phased array coil demonstrated slightly worse shimming effect larger FWHM; Fig 4). The discrepancy of the line width caused by different coils was about 0.8 Hz (0.00625 ppm). No significant differences on water suppression were observed (*P*>0.05, Table 1).

**Fig 5:**
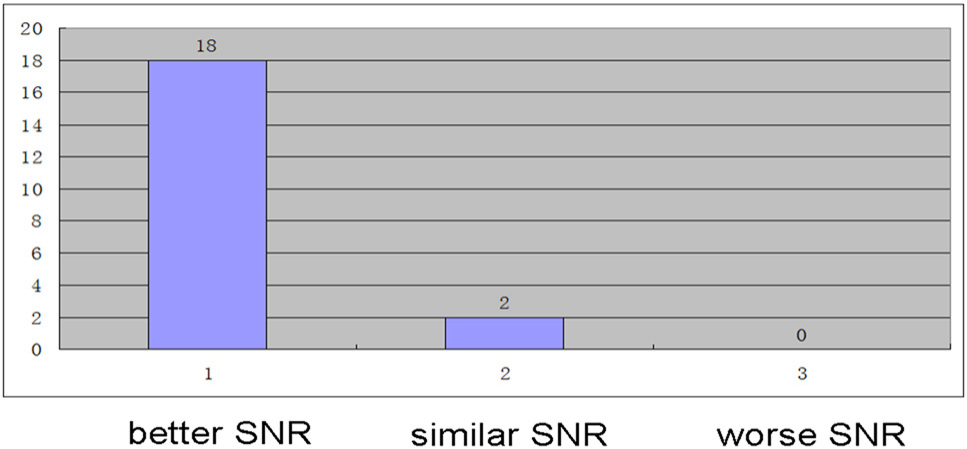
The group using phase array coil demonstrated better SNR.

Comparing the paired spectra from the two coils, the group using the phased array coil demonstrated better SNR than the group using body coil (*Z* = −4.243, *P*<0.001). Among the obtained spectra, 2 pairs were marked 0, 18 were marked 1, 0 was marked −1 (Fig 5). The results showed no significant differences on the major liver quantitative information of Cho/Lip2 height, Cho/Lip2 area, and lipid content on a 3.0T system (All *P*>0.05, Table 1).

### Part 2 Calculation of homogeneity and SNR of body and phased array coils for theoretical analysis

**Fig 6:**
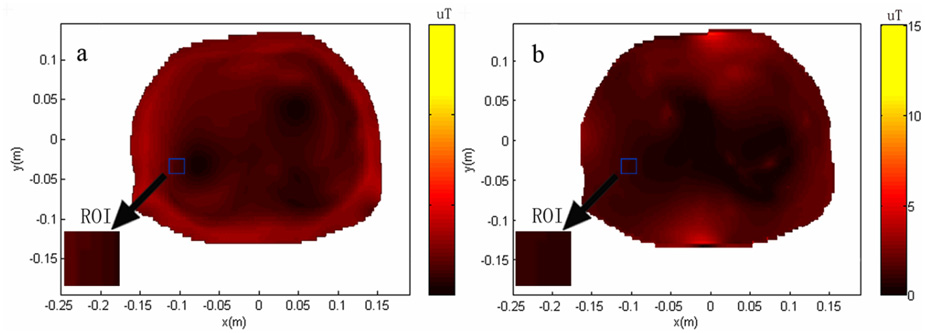
Theoretical B_1_ field distribution through the transverse planes centered at the ROI used for the calculation of *RSD* of different coils. (a) transverse plane of body coil, the *RSD* was 6.14% within the ROI, indicating the homogeneity of 93.86%; (b) transverse plane of phased array coil, the *RSD* was 19.90% within the ROI, indicating the homogeneity of 80.10%. The less the *RSD* means the more homogeneous B_1_ field. The results showed that the homogeneity of the body coil is better than that of phase array coil.

**Fig 7:**
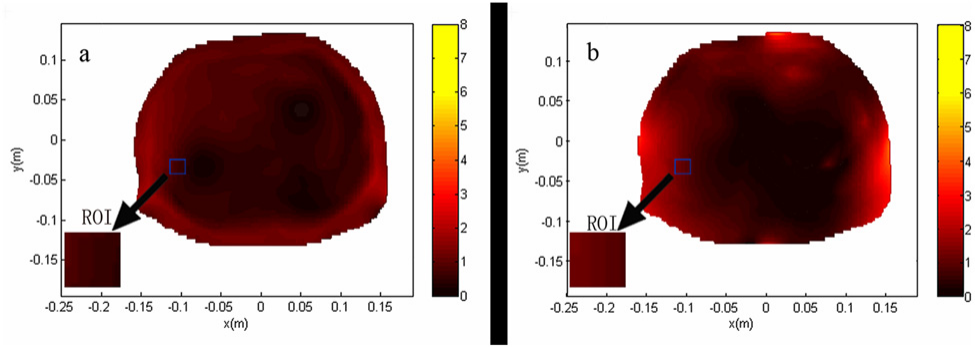
Theoretical B_1_ field distribution through the transverse planes centered at the ROI used for the calculation of SNR of different coils. The value of SNR was obtained when the mean B_1_ value within the ROI was 1.957 μT. The SNR of body coil was normalized to 1 within the ROI for the transverse plane of body coil (a). The SNR of the transverse plane of phased array coil was 2.8387 (b). The results showed that the SNR of the phase array coil is higher than that of the body coil.

The obtained theoretical B_1_ field distributions used for the calculation of *RSD* and SNR of different coils are shown in Figs 6 and 7.

Both transverse planes that pass through the center of ROI were selected to show the distribution. Inside the ROI, the *RSD* of the body coil was 6.14%, indicating the homogeneity of 93.86% (Fig 6A); the *RSD* of the phase array coil was 19.90%, indicating 80.10% homogeneity (Fig 6B). Less *RSD* means more homogeneous B_1_ field. The results showed that the homogeneity of the body coil was better than that of the phase array coil.

The SNR of the body coil was normalized as 1 inside the ROI; the SNR of phase array coil was 2.8387 (Fig 7). The results showed that the SNR of the phase array coil is better than that of the body coil.

## Discussion

For the past decade, only few researches in improving the technology to develop applications within the abdomen and better understand the utility of MRS for malignancy detection have been conducted. The recent installation of multicoil arrays for the body offers new opportunities for performing hepatic MRS. Given that spectroscopy at 3.0T provides improved *SNR* and spectral resolution compared with 1.5T MRI scanner, it is expected to yield more reliable measurements of metabolite concentrations [17–21]. RF coil serves as the immediate interface between the complex chain of MRS hardware and the patient; hence, their performance characteristics are crucial in the determination of image quality as measured by the SNR and radiofrequency field homogeneity. Therefore, assessment of the spectral quality of hepatic proton MRS at 3.0T between the body coil and the phased array coil is very important [10,12].

Each MRS technique has advantages and disadvantages; hence, choosing the right technique for a specific purpose is important to improve the quality of the results. Our previous studies indicated that, although we used a 3.0 T MR Imager and shorter TE to increase SNR, spectra containing only noise without any identifiable choline metabolite peaks still existed in few cases [22]. As a result, the short TE was selected to maximize detection of choline in this study. Breathhold imaging has been proven to be far more satisfactory, but imposes a patient-dependent time limit on the acquisition [18, 19]. We tried to look for a compromise between SNR and acquisition time. The measurement parameters used were 1500 msec (TR) and 64 signal acquisitions (total acquisition time was 2 minutes 12 seconds) in our research.

Linewidth is usually defined independent of the lineshape as the full-width at half-maximum peak height in the frequency domain. It determines the capability of MRS to discern spectral features. As shimming improves the field homogeneity, linewidths become smaller and the spectroscopy resolution is enhanced. The line width of a spectral peak is dependent both on the intrinsic T2 of the metabolite and the homogeneity of the magnetic B_1_ field in the region. Comparing the paired spectra from the two coils, our results suggest that the group using the body coil demonstrated slightly better shimming effect. The discrepancy of the line width caused by different coil was about 0.8 Hz (0.00625 ppm). The result of the clinical study was consistent with that of the theoretical calculation. In this study (Part 2), our results showed that, inside the ROI, the *RSD* of the body coil was 6.14% and that of the phase array coil was 19.90%, indicating the homogeneity of 93.86% and 80.10%, respectively. The homogeneity exceeding 70% is considered qualified for a modern high-resolution NMR probe-head [5, 12, 23].

SNR is often defined as the height of the largest metabolite peak divided by the root mean square of the amplitude of the noise in a signal and artifact-free part of the spectrum. Improved SNR is achieved by making a number of technical adjustments, such as using a larger ROI, increasing the total acquisition time, using a high-field strength magnet, or using a local receiving coil. The body coil provides homogeneous transmission and reception over a large anatomic region. However, given that the noise reception is nonlinearly proportional to the volume of tissue being interrogated, the overall SNR of the body coil is lower than that of the phased array coil. In this study, the SNR of the body coil was normalized to 1 within the ROI for the transverse plane of the body coil, while the SNR of the transverse plane of phased array coil was 2.8387. The SNR for liver metabolites is low because of their low concentration and the significant distance from the RF coils. The phased array coil can obtain images with higher SNR compared with those by body coil, which is more suitable for 3.0T breathhold hepatic proton MRS. [2,5,13,15]

MR spectroscopy may be used to quantify liver fat by measuring lipid peaks, as well as diagnose malignancy, usually by measuring the choline peak (Cho). The signals of the corresponding lipid groups can be observed at 1.3 ppm (Lip2) for (-CH2) and 0.9 ppm for (-CH3), as well as between 2.0 and 2.3 ppm for (-CH = CH-CH2-), with significantly lower intensity signals. In this study, analysis of the spectra only focused on the concentrations of Lip2 and Cho. Absolute quantification of choline is impractical for most clinical applications. A few studies of in vivo MR spectroscopy reported an increase in Cho/Lip2 within tumors such as hepatocellular carcinoma and a reduction in Cho/Lip2 after transarterial embolization were performed for hepatocellular carcinoma. Meanwhile, MRS is effective for quantifying liver fat [3, 5, 6, 21, 24–26]. Therefore, we focused on Cho/Lip2 height, Cho/Lip2 area, and lipid content between the body coil and the phased array coil.

Some authors have argued that the body coil is more suitable for *in vivo* hepatic MRS acquisition. F. Fischbach and coworkers considered that the body coil provides a more homogeneous B1 field with respect to quantitation compared with the surface coil, thereby facilitating data comparison [17]. We view these effects of shimming as clinically unimportant because overlap is much more uncommon when quantifying the peaks of metabolites of the liver. The phased array coil can obtain images with higher SNR compared with those by body coil, which is more suitable for 3.0 T hepatic proton MRS. Knowledge on these findings is helpful to clinicians.

This technique has its limitations in methodology. Although a 3.0 T MR imager and shorter TE were used to increase SNR, spectra containing only noise without any identifiable choline metabolite peaks still existed in certain cases. Advanced techniques, such as parallel transmission, may demonstrate improved SNRs and spectral resolution between the MRS peaks. Application of these new techniques may be necessary to answer this question [27–29]. In addition, the data reported in this study were only derived from normal individuals, without using patients with hepatitis, cirrhosis, fatty liver, and hepatocellular carcinoma. The actual purpose of the preferential use of the phased array system and its clinical application for diagnosis of liver diseases remains unclear. It can only be speculative based on the results presented in this for clinical use. A further study is required.

In conclusion, compared with the body coil group, the phased array group had slightly worse shimming effect and better SNR. The discrepancy of the line width caused by different coils was about 0.00625 ppm. No significant differences on the major liver quantitative information of Cho/Lip2 height, Cho/Lip2 area, and lipid content were observed. The theoretical analysis and clinical experiment showed that the phased array coil was superior to the body coil with respect to 3.0 T breathhold hepatic proton MRS.
